# Supervised machine learning models applied to disease diagnosis and prognosis

**DOI:** 10.3934/publichealth.2019.4.405

**Published:** 2019-10-17

**Authors:** Maria C Mariani, Osei K Tweneboah, Md Al Masum Bhuiyan

**Affiliations:** 1 Department of Mathematical Sciences, University of Texas, El Paso, United States; 2 Computational Science Program, University of Texas, El Paso, United States

**Keywords:** machine learning, supervised learning, predictive models, breast cancer, heart disease

## Abstract

This work analyses the diagnosis and prognosis of cancer and heart disease data using five Machine Learning (ML) algorithms. We compare the predictive ability of all the ML algorithms to breast cancer and heart disease. The important variables that causes cancer and heart disease are also studied. We predict the test data based on the important variables and compute the prediction accuracy using the Receiver Operating Characteristic (ROC) curve. The Random Forest (RF) and Principal Component Regression (PCR) provides the best performance in analyzing the breast cancer and heart disease data respectively.

## 1. Introduction

Machine learning (ML) algorithms help to detect and classify chronic diseases like cancer, heart disease, tumor, diabetes, and several others [1]–[5]. These techniques are useful in many statistical and optimization problems that allow computers to learn past observations and to detect patterns in new set of observations. In addition, some of the ML techniques help to identify the sources and order variables that plays significant role in the cause of the disease.

In the past decades, breast cancer (BC) has a high mortality rate [6]. In the United States, the risk of a woman having breast cancer at some point in her life is about 12%. In fact, one in eight women has the risk of developing breast cancer in her life [6]. Almost (80%) breast cancers are invasive and they break through the walls of the glands or ducts where they appear and then spread into surrounding breast tissue. However, the mortality rate has declined over the past years with the application of ML techniques. A recent analysis shows that the survival rate is 91% after 5 years of diagnosis and 80% after 15 years of diagnosis [6]. *Vikas* et al. compares several supervised learning classifiers, such as Naive Bayes, Support Vector Machine-Radial Basis Function (SVM-RBF) kernel, Radial Basis Function neural networks, and Decision trees to find the best classifier in breast cancer datasets [7]. Their studies show that SVM-RBF kernel is more accurate (96.84%) than the other classifiers. T. *Joachims* et al. shown (95.06%) of accuracy with the use of neuron-fuzzy techniques [7]. Other researchers have worked on several data mining algorithms such as SVM, k-nearest neighbor algorithm (IBK), and Bloom Filter (BF) Tree on breast cancer data [8].

Heart disease has created grave concerns among medical researchers. A recent analysis has shown that every year about 735,000 Americans have heart attacks. Of these, 525,000 are first heart-attack victims and 210,000 have already had heart attacks [9]. One of the major challenges in heart disease is its correct detection inside a human. Since a lot of parameters and technicality are involved for accurately predicting this disease, there is a vast scope of research including ML algorithms. In ref. [10], the authors used logistic regression, artificial neural network, and support vector machine techniques on heart disease data and concluded the highest classification 85% of accuracy with logistic regression model. Other authors also worked on artificial neural network and fuzzy neural network on Cleveland heart disease data and obtained 86.8% of accuracy [11]. Thus ML techniques play a crucial role in the diagnosis and prognosis of heart disease in patients.

In previous studies involving the analysis of BC and heart disease data, even though the ML algorithms provided good prediction accuracy, most of the models lack good interpretations. An important feature of classification problem is that the model should be able to order key variable that causes the disease. Therefore to overcome some of the drawbacks in the previous studies, we analyze the BC and heart disease data using five ML models that have good prediction accuracy and the results obtained are easy to interpret. The models used are as follows: Logistic regression, Principal component regression, Random Forest, Multivariate adaptive regression splines and Support vector machine for breast cancer and heart disease diagnosis and prognosis. The objective of this study is to firstly predict the Class variable and to conclude whether a patient's tumor is malignant or benign and secondly to determine the attributes which are important to help detect that a patient suffering from cancer.

The paper is organized as follows: The methodology section presents a brief background of the five supervised machine learning techniques. The regularization techniques to fit the best predictive model are also discussed. In the data background section, we present a brief description of the datasets used in this studies. We discuss the association of the datasets in the exploratory data analysis section. Applications of the models to cancer and heart disease data are presented in the results and discussion section. In the last section, we present the conclusion and discussion the suitability of our models to diagnose the cancer and heart disease data.

## 2. Methodology

This section briefly describes the five machine learning techniques that will be used to analyze breast cancer and heart disease data. We will discuss the regularization techniques to obtain the best model with important features in the datasets. We will also estimate the tuning parameters of the models to obtain the lowest mean squared error, lowest miss-classification rate and highest predictive accuracy. We begin with the logistic regression model.

### 2.1. Logistic regression model

Logistic regression is a powerful classification algorithm that is used to predict the probability of a categorical variable. We assume that the predictors (*x_k_*) are independent of each other, so the model has no multicollinearity. We express the model as:

$$\text{logit}\left(p\right)=\beta_{0}+\beta_{1}x_{1}+\beta_{2}x_{2}+\beta_{3}x_{3}+\cdot \cdot \cdot +\beta_{k}x_{k}$$ (1)

where *p* is the probability of presence of the characteristic of interest and *β*_0_, *β*_1_, …, *βk* are the coefficient parameters. The logit transformation is defined as the log odds that is $ln\frac{p}{1-p}$. So the logistic regression model is similar to a linear regression, but it is constructed using the natural logarithm of the odds of the target variable. Thus:

$$ln\frac{p}{1-p}=\beta_{0}+\beta_{1}x_{1}+\beta_{2}x_{2}+\beta_{3}x_{3}+\cdot \cdot \cdot +\beta_{k}x_{k}$$ (2)

Since logistic regression predicts probabilities, rather than classes, we can fit it into the data using the likelihood technique. The likelihood helps to find the best model that explains the datasets well. The dataset used in this study contains a vector of features (*x_i_*) and an observed class (*y_i_*). We assume the probability of that class is either *p*, when *y_i_* = 1, or 1−*p*, when *y_i_* = 0. So the likelihood function of Eq 2 is as follows:

$$L(\beta_{0}+\beta_{1}x_{1}+\cdot \cdot \cdot +\beta_{k}x_{k})=\overset{N}{\underset{i=1}{\prod }}p{\left(x_{i}\right)}^{y_{i}}{\left(1-p(x_{i})\right)}^{1-y_{i}}$$ (3)

We now seek to estimate the parameters *β*_0_, *β*_1_, …, *βk* that maximize the likelihood function *L*(*β*_0_ + *β*_1_*x*_1_ + … + *β_k_x_k_*) in Eq 3. We maximize the logarithm of the likelihood function [12] as follows:

$$l(\beta )=\overset{N}{\underset{i=1}{\sum }}(y_{i}\beta^{T}x_{i}-log(1+e^{\beta^{T}x_{i}}))$$ (4)

We then use some regularization techniques to obtain a parsimonious model with important features from the original model. The regularization technique penalizes the magnitude of coefficients of features thereby minimizing the error between predicted and actual observations. In this study, the regularization techniques used in the logistic regression are the *L*_1_ and *L*_2_ regularization.

#### 2.1.1. *L*_1_ regularization

We use the least absolute shrinkage and selection operator (lasso) regularization technique by adding an *L*_1_ penalty term in Eq 4. This forces the absolute value sum of the regression coefficient to be less than a fixed value. This is due to the fact that the tuning parameter makes certain coefficients to be set to zero, effectively by choosing a simpler model that does not include those coefficients [13]. So we maximize the penalized versions as follows:

$$l_{\lambda }(\beta )=\overset{N}{\underset{i=1}{\sum }}(y_{i}\beta^{T}x_{i}-log(1+e^{\beta^{T}x_{i}}))-\lambda \overset{p}{\underset{j=1}{\sum }}|\beta_{j}|$$ (5)

where *λ* is a tuning parameter that controls the strength of penalty term. The parameter *λ* is selected in a way that the resulting model minimizes the out of sample error.

#### 2.1.2. *L*_2_ regularization

We also use Ridge-regression by adding a *L*_2_ penalty term in Eq 4. This regularization technique overcomes the multicollinearity problem in our data. When we have multicollinearity in the data, the variance of estimation goes to large values. So the parameter estimation may be far from the true value. To overcome this issue, we add a degree of bias to the regression estimates and shrink the estimators to the true parameters. We maximized the *L*_2_ penalized versions [14] as follows:

$$l_{\lambda }\left(\beta \right)=\overset{N}{\underset{i=1}{\sum }}(y_{i}\beta^{T}x_{i}-log\left(1+e^{\beta^{T}x_{i}}\right)-\lambda \overset{p}{\underset{j=1}{\sum }}\beta_{j}^{2}$$ (6)

where *λ* controls the amount of regularization.

### 2.2. Principal component regression

Principal Component Regression (PCR) is based on the principal components of data. We perform PCR technique by transforming the independent variables (*X*) to their principal components (PC) of data. An important feature of principal components is that they reduce the dimension of dataset that explain most of the variability of the original data [15]. The regression equation can be expressed as:

$$\underline{Y}=XB+\epsilon$$ (7)

where *Y* is the standardized dependent variable, *X* is the standardized independent variables, *B* is the regression coefficients to be estimated and *ϵ* is the residual term. We now estimate the regression coefficients using ordinary least square method as follows:

$$\hat{B}={\left(X^{\prime }X\right)}^{-1}X^{\prime }Y$$ (8)

Since the variables are standardized, *X*′*X* is a correlation matrix of independent variables from the data. In order to perform the PCR, we transform the independent variables to their principal components as follows:

$$X^{\prime }X=PDP^{\prime }=Z^{\prime }Z$$ (9)

where *D* is a diagonal matrix of the eigenvalues of *X*′*X*; *P* is the eigenvector matrix of *X*′*X* where *P* is an orthogonal matrix, that is *P*′*P* = *I*, where *I* is an identity matrix. Now *PDP*′ gives us the new matrix of principal components (*Z*′*Z*). The new variables *Z* are the weighted averages of the original variables *X*. Since these new variables are principal components, their correlations with each other would be all zero. We then regress *Y* on *Z* obtaining least squares estimates of *A*. The estimation formula is as follows:

$$A={\left(Z^{\prime }Z\right)}^{-1}Z^{\prime }Y=D^{-1}Z^{\prime }Y$$ (10)

### 2.3. Random forest

The random forest technique is a type of additive model that predicts the data by combining decisions from a sequence of base models. It reduces the variance by avoiding over fitting of the model. The class of base models can be expressed as follows:

$$g\left(x\right)=f_{0}\left(x\right)+f_{1}\left(x\right)+{\text{f}}_{2}\left(x\right)+\cdot \cdot \cdot$$ (11)

where the final model *g* is the sum of simple base models *f_i_*. We define each base classifier as a simple decision tree. So it is an ensemble technique that considers multiple learning algorithms to obtain best predictive model. At this point, all the base models or trees are made independently using a different subsample of the data. Once we have a new generated training set, we divide it randomly into two parts. The two-third samples are used to build a tree and the one-third samples are used to obtain the predictions of trees. We take the majority vote of these one-third predictions as the predicted value for the data point and then we estimate the error. For a full detail study of Random Forest, the reader is referred to the reference in [16]. We now present the algorithm of random forest that is used in this study:

1. We first take a random sample of size N with replacement from the data.
2. Take a random sample without replacement of the predictors.
3. Construct a split by using predictors selected in step 2.
4. Repeat steps 2 and 3 for each subsequent split until the tree is as large as desired.
5. Drop the out-of-bag data down the tree. We then store the class assigned to each observation along with each observation's predictor values.
6. Repeat steps 1–5 for large number of times.
7. For each observation in the dataset, we count the number of trees that it is classified in one category over the number of trees.
8. Assign each observation to a final category by a majority vote over the set of trees. Thus, if 51% of the time over a large number of trees a given observation is classified as a “1” that becomes its classification.

The random forest includes three main tuning parameters such as node size, number of trees (ntree) and number of predictors sampled (mtry) for splitting. To build a best predictive model, we estimate the best tuning parameters and important variables using mean decrease accuracy (MDA) and mean decrease Gini (MDG) indices. The MDA determines the importance of a variable by measuring the change in prediction accuracy, when the values of the variable are randomly permuted compared to the original observations. However, the MDG index is a measure of how each variable contributes to the homogeneity of the nodes and leaves in the resulting random forest. For the details of these methodologies, consult [17] and references therein.

### 2.4. Multivariate Adaptive Regression Splines (MARS)

MARS is a non-parametric regression technique. It is used to study the nonlinear relationship between a target variable and a set of predictors with the help of splines. We partition the training data set into piecewise linear segments with different gradients. These segments are connected smoothly together and we define them as splines [18]. The connection points between the segments are known as knots. An important property of MARS model is that it makes no assumptions about the underlying functional relationships between dependent and independent variables. We now define the MARS model as follows:

y=f(x)+ϵ (12)

where *y* is a response variable, *f*(*x*) is a “true” underlying function, *x* = (*x*_1_, *x*_2_, …, *x_p_*)*^T^*, and epsilon is the error term. To approximate a nonlinear relationship, we first develop a flexible model using piecewise linear basis functions (BFs) as:

$${\left(x-t\right)}_{+}=\{\begin{array}{ll} x-t, & \text{if }x>t\\ 0 & \text{if otherwise} \end{array}\text{ and }{\left(t-x\right)}_{+}=\{\begin{array}{ll} t-x, & \text{if }x<t\\ 0 & \text{if otherwise} \end{array}$$ (13)

At this point, *f*(*x*) can be expressed as a linear combination of BFs and their interactions:

$$f\left(x\right)=\beta_{0}+\overset{M}{\underset{m=1}{\sum }}\beta_{m}\lambda_{m}\left(X\right)$$ (14)

where each *λ_m_* is a BF, *β_i_* is constant coefficient that is estimated through a least-squares method. In order to fit the MARS model with the data, we perform forward stepwise technique on the training dataset with initialize value of *M* = 0, *λ*_0_(*x_i_*) = 1, and *f*(*x_i_*) = *β*_0_*λ*_0_(*x_i_*). At each subsequent step, we add the basis pair that gives the maximum reduction in the training error. This process of adding BFs continues until the model reaches some predetermined maximum number. The forward stepwise process over fits the model. We improve the model using backward deletion technique. This technique keeps deleting the less significant terms of the model to find best sub-model. We compare the sub-models using Generalized Cross-Validation (GCV) to obtain the best one. The GCV is the mean-squared residual error divided by a penalty that is dependent on the model complexity [19] i.e.:

$$GCV=\frac{\frac{1}{N}\sum_{i=1}^{N}{\left[y_{i}-f(x_{i})\right]}^{2}}{{\left[1-\frac{M+d\times (M-1)/2}{N}\right]}^{2}}$$ (15)

where *M* is the number of BFs, *d* is a penalty for each basis function included in the developed submodel, *N* is the number of data sets, and *f*(*x_i_*) denotes the MARS predicted values. So the numerator term is the mean square error of the evaluated model, that penalized by the denominator. In this study, we make the order of important variables by analyzing the variance (ANOVA) between the BFs involving one variable and the BFs involving pairwise interactions. For the details of this methodology, please see the reference in [16].

### 2.5. Support Vector Machine (SVM)

SVM is a supervised ML technique that is used for both classification and regression tasks. It identifies the optimal decision boundary that separates data points from different classes, and then it predicts the class of test observations using the separation boundary [20]. So we first normalize and scale the variables to obtain a decision boundary as follows:

$$y_{i}\left(\beta^{T}x_{i}+\beta_{0}\right)\geq M\left(1-\xi_{i}\right)\text{for},i=1,\cdot \cdot \cdot ,n.$$ (16)

which is a non-convex programming problem which computes the distance from a wrongly classified observation *x_i_* to its corresponding margin. Here, *ξ_i_* ≥ 0 measures the degree of missclassification of the *i*-th individual. We now consider the problem as follows:

$$\underset{\beta ,\beta_{0},M}{\text{max}}M;\text{ subject to }\left|\left|\beta \right|\right|=1,\text{ and }y_{i}\left(\beta^{T}x_{i}+\beta_{0}\right)\geq M\left(1-\xi_{i}\right)$$ (17)

where $\overset{n}{\underset{i=1}{\sum }}\xi_{i}\leq C$ and the constant C determines the possible miss classification rate. The higher the value of C, the less likely it is that the SVM algorithm will miss classify a point. So in this study our approach is to tune the parameter *C*. Now we drop the constraint ║β║ = 1 and set *M* = 1/║β║ in the problem and we obtain:

$$\begin{array}{l} \begin{array}{rr} \underset{\beta ,\beta_{0},}{\text{min}}\frac{1}{2}||\beta |{|}^{2} & +C.\overset{n}{\underset{i=1}{\sum }}\xi_{i},\\ \text{s}.\text{t}.\xi_{i}\geq 0 & \text{ and }y_{i}(\beta^{T}x_{i}+\beta_{0})\geq 1-\xi_{i} \end{array} \end{array}$$ (18)

The above equation is a convex programming problem and can be solved via Lagrangian and Karush-Kuhn-Tucker (KKT) conditions as follows:

$$ℒ(\beta ,\beta_{0},\xi ,\alpha ,\gamma )=\frac{1}{2}||\beta |{|}^{2}+C\overset{n}{\underset{i=1}{\sum }}\xi_{i}-\overset{n}{\underset{i=1}{\sum }}\alpha_{i}\{y_{i}(\beta^{T}x_{i}+\beta_{0})-(1-\xi_{i})\}-\overset{n}{\underset{i=1}{\sum }}\gamma_{i}\xi_{i}.$$ (19)

Setting $\frac{\partial ℒ}{\partial \beta },\frac{\partial ℒ}{\partial \beta_{0}},\frac{\partial ℒ}{\partial \xi_{i}}$ to be 0 gives the following conditions:

$$\hat{\beta }=\overset{n}{\underset{i=1}{\sum }}\alpha_{i}y_{i}x_{i},\underset{i=1}{\sum }\alpha_{i}y_{i}=0,\text{ and }\alpha_{i}+\gamma_{i}=C,\forall i$$ (20)

where, *α_i_* ≥ 0, *γ_i_* ≥ 0, and *ξ_i_* ≥ 0. We can solve the Lagrangian problem via coordinate descent method. Now the KKT complementary slackness condition yields the following case:

$$\{\begin{array}{l} \text{ If }\alpha_{i}=0,y_{i}(\beta^{T}x_{i}+\hat{\beta_{0}})\geq 1\\ \text{ If }\alpha_{i}=C,y_{i}(\beta^{T}x_{i}+\hat{\beta_{0}})\leq 1\\ \text{ If }0<\alpha_{i}<C,y_{i}(\beta^{T}x_{i}+\beta_{0})=1 \end{array}$$ (21)

In this study, we compute the SVM for different values of *C* and choose the optimal *C* that maximizes the model cross-validation accuracy. We use the caret package in R software to compute the linear SVM, radial SVM techniques. For more details of the SVM technique, the reader is referred to [13] and references therein.

### 2.6. Data background

In this study, we analyzed the breast cancer [10] and heart disease [11] datasets, that are available in UCI machine learning repository. The breast cancer datasets has 699 observations with 11 variables. Table 1 summarizes the short description of the breast cancer dataset. The “Class” column is the response variable that includes the status of a tumor as malignant (breast cancer) or benign (not breast cancer). Our objective is to predict the “Class” variable and to conclude whether a patient's tumor is malignant or benign.

**Table 1.** Background of cancer data.

Variables	Coefficients
Id	Sample code number
Cl. thickness	Clump Thickness
Cell. size	Uniformity of Cell Size
Cell. shape	Uniformity of Cell Shape
Marg. adhesion	Marginal Adhesion
Epith. c. size	Single Epithelial Cell Size
Bare. nuclei	Bare Nuclei
Bl. cromatin	Bland Chromatin
Normal. nucleoli	Normal Nucleoli
Mitoses	Mitoses
Class	Class

For the heart disease data, we used 14 variables that play important role in causing heart disease. The variables are such as age of patients, sex, chest pain, resting blood pressure, fasting blood sugar, number of major vessel and several others. Table 2 summarizes the short description of heart disease dataset. The target variable is *num* that contains the rate of diameter narrowing of coronary artery. It takes value 0 when the rate < 50% and value 1 when the rate > 50% We assume that the patient has no heart disease when *num* is 0 and the patient has heart disease when *num* is 1. The goal is to predict the *num* variable using the ML techniques to determine whether a patient has heart disease.

**Table 2.** Background of heart disease data.

Variables	Coefficients
Age	Age of patient
sex	Sex, 1 for male
cp	chest pain
trestbps	resting blood pressure
chol	serum cholesterol
fbs	fasting blood sugar larger 120mg/dl (1 true)
restecg	resting electroc. result (1 anomality)
thalach	maximum heart rate achieved
Exang	exercise induced angina
Oldpeak	ST depression induced by exercise relative to rest
Slope	the slope of the peak exercise ST segment
ca	number of major vessel
Thal	Thalassamia
num	angiographic disease status

In the next section, we perform some exploratory data analysis by studying the association and correlation of all variables in the data sets, which are effective in capturing the characteristic and patterns of the data.

## 3. Exploratory data analysis

**Figure 1.**
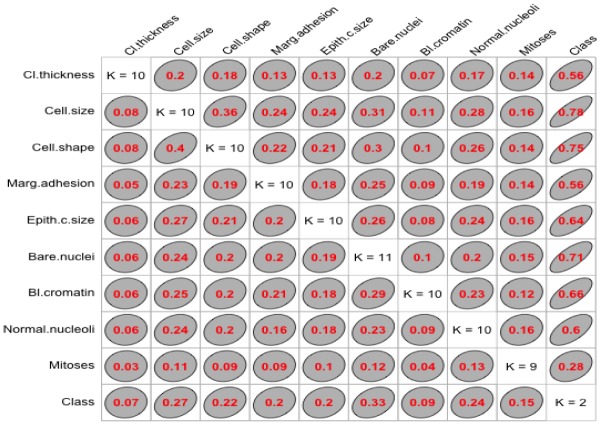
Association among the cancer variables.

**Figure 2.**
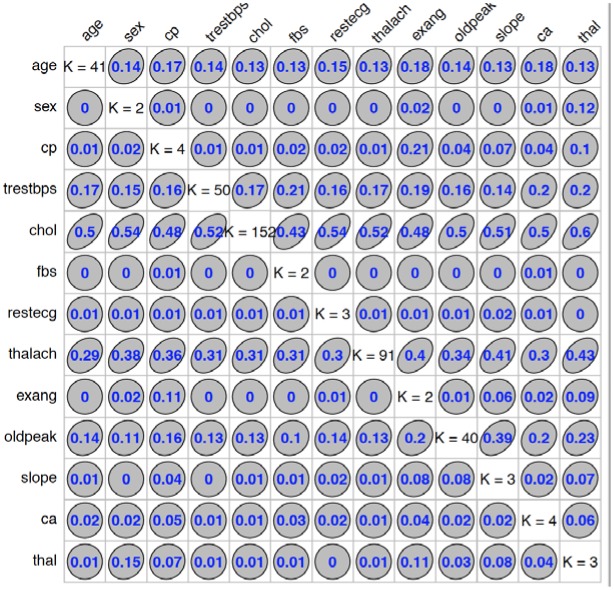
Association among the heart disease variables.

We used “GoodmanKruskal” library in R program to determine the association among predictors. Figures 1 and 2 represent the association among the variables for cancer and heart disease data, respectively. In these figures, the diagonal element *K* refers to the number of unique levels for each variable. The measure of association indicates the strength of the relationship among the variables. The off-diagonal elements contain the forward and backward measures (*τ*) for each variable pair. In Figure 1, we observe that the variable Cell.size (*x*) is almost perfectly predictable (i.e. *τ*(*x*,*y*) = 0.78) from Class (*y*) and this forward association is quite strong. On the other hand, the reverse association between Class and Cell.size (*τ*(*x*,*y*) = 0.27) is not strong association as forward. Similarly, we can analyze the forward and backward association among the variables in heart disease data (see Figure 2).

## 4. Results and discussions

In this section we present the results of the machine learning techniques when they are applied to the breast cancer and heart disease datasets. We trained five ML models to accurately predict whether a sample patient has been diagnosed with heart disease or cancer. We randomly split the data into training set (67% for building a predictive model) and test set (33% for evaluating the model). We then compute the prediction mean squared error (PMSE), missclassification rate (MCR), and prediction accuracy using the ROC curve that validates the fitted model with the data. The analyses was done by R statistical programs.

### 4.1. Analysis of fitted models

#### 4.1.1. Logistic regression

We first used the logistic regression technique for the train data to build a predictive model. In this case, we used the lasso regularization with *L*_1_ penalty and obtained the tuning parameter *λ* with cross-validation. The *L*_1_ penalty is used for both variable selection and shrinkage, since it has the effect of forcing some of the coefficient estimates to be zero. Tables 3 and 4 represent important predictors using the best predictive model with *L*_1_ penalty. It is clear that *Cell.size* for cancer disease (Table 3) and *Age*, *Sex*, *Trestbps*, *Chol*, *Fbs* and *Restecg* for heart disease (Table 4) are not important predictors. We then predict the test data using this predictive model and compute the accuracy.

**Table 3.** Coefficients of important predictors using LGR(*L*_1_) model for Cancer data.

Variables	Coefficients
Cl.thickness	− 0.4891
Cell.size	0.0000
Cell.shape	− 0.2656
Marg.adhesion	− 0.3596
Epith.c.size	− 0.2128
Bare.nuclei	− 0.2988
Bl.cromatin	− 0.3582
Normal.nucleoli	− 0.1435
Mitoses	− 0.30637

**Table 4.** Coefficients of important predictors using LGR(*L*_1_) model for heart data.

Variables	Coefficients
Age	0.0000
Sex	0.0000
Cp	0.2847
Trestbps	0.0000
Chol	0.0000
Fbs	0.0000
Restecg	0.0000
Thalach	− 0.0073
Exang	0.4792
Oldpeak	0.1605
Slope	0.2172
Ca	0.4164
Thal	0.3021

We also used the Lasso regression model with *L*_2_ penalty term on the parameters *β*. The reason is that *L*_2_ penalty overcomes the multicollinearity issue of the datasets. We tuned *λ* until we find a model that generalizes well to the test data. In this case, we select the tuning parameter by 10-fold cross validation. From Table 5, we see that the prediction mean squared error and classification rate of this model are very low for both cancer and heart disease data.

#### 4.1.2. Principal component regression

We used a dimension reduction tool, namely principal component regression to reduce the set of correlated predictors. In this case, we transformed the entire dataset into three principal components to build the predictive model. The first principal component contains most of the variability in the data. PCR also overcomes the multicollinearity issue of the cancer and heart disease datasets. We compared the predictive performance of PCR to the other methodologies in Table 5.

#### 4.1.3. Random forest

**Figure 3.**
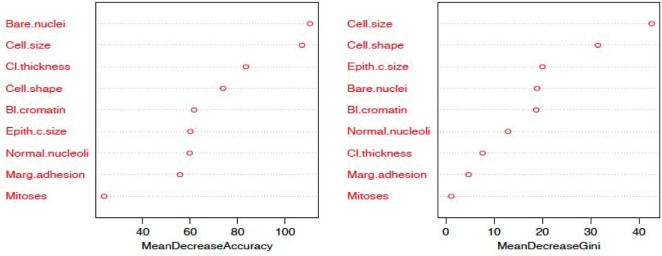
Variable importance plot using random forest model for cancer data.

**Figure 4.**
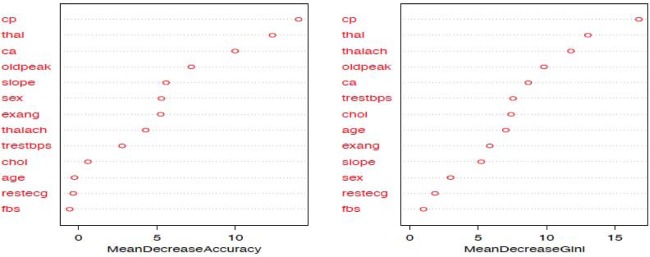
Variable importance plot using random forest model for heart-disease data.

The random forest model is fit with the train data to build a predictive model. We used 500 trees and sampled 3 variables at each split. We obtained a very good prediction accuracy on test data, which are 97.38% for cancer disease, and 88.10% for heart disease. We also ranked the variable importance using the Mean Decrease Accuracy and Mean Decrease Gini indices. From Figure 3, the predictor *Cell.size* is the most important variable and the predictor *Mitoses* is the least important variable in causing breast cancer using the Mean Decrease Gini index. From Figure 4, we see that the predictor *Cp* is the most important predictor and the predictor *Fbs* is the least important predictor in causing heart disease.

#### 4.1.4. Multivariate adaptive regression splines

**Figure 5.**
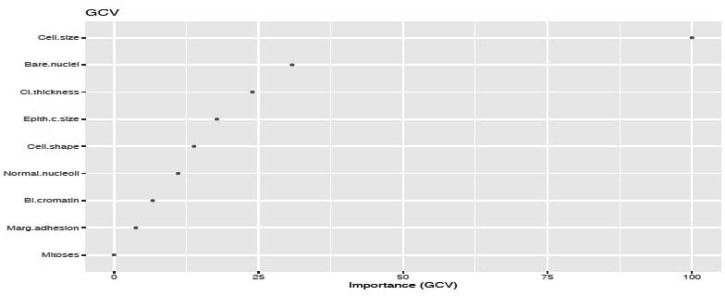
Variable importance plot using mars model for cancer data.

**Figure 6.**
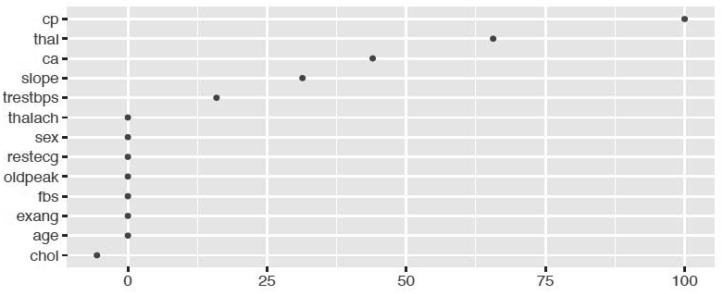
Variable importance plot using MARS model for heart disease data.

We fitted the MARS model with the train data using 3-fold cross-validation. The reason of using MARS model is that it does not assume or impose any particular type or class of relationship between the predictor variables and the target variable. We ranked the predictors in terms of importance using the Generalized Cross-Validation (GCV) (see Figures 5 and 6). The GCV is a type of regularization technique that trades-off goodness-of-fit against the model complexity. It adjusts the training residual sum of squares (RSS) and takes into account the flexibility of the model. In Figures 5 and 6, we see that *Cell.size* and *Cp* are the most important variables for breast cancer and heart disease, respectively, which are consistent with the results obtained using logistics regression and random forest.

#### 4.1.5. Support vector machine

We analyzed two types of support vector machine technique namely, SVM linear and SVM kernel. To fit the models, we first standardized the data and used 10-fold cross-validation in training data. We evaluated different cost levels (C) to obtain the best predictive model with optimal cost. For SVM linear, we obtained the highest accuracy of predictive model when costs are 0.05 (cancer data) and 0.04 (heart disease data). On the other hand, for Kernel SVM, we achieved the highest accuracy when *γ* is 0.001 (cancer data) and 0.002 (heart disease data). In Table 5, we summarize the predictive mean square error (PMSE) for the predicted probabilities and missclassification rate (MCR) for both cancer and heart disease data.

**Table 5.** Model evaluation.

Models	Cancer Data		Heart-disease Data	
	PMSE	MCR	PMSE	MCR
LGR-L_1	0.0306	0.0284	0.1512	0.1720
LGR-L_2	0.0349	0.0349	0.1491	0.1935
PCR	0.0384	0.0349	0.1078	0.1182
RF	0.0205	0.0262	0.1447	0.1720
MARS	0.0203	0.0305	0.1588	0.2043
SVM-linear	0.0305	0.0305	0.1935	0.1935
SVM-nonlinear	0.0219	0.0305	0.1445	0.1627

#### 4.1.6. Model accuracy

**Figure 7.**
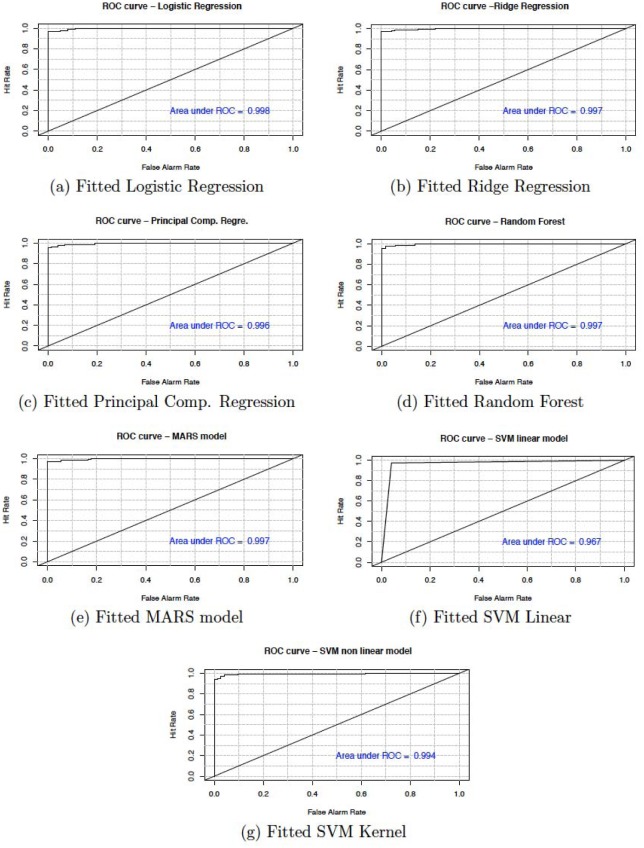
Model evaluation using ROC curve for cancer data.

**Figure 8.**
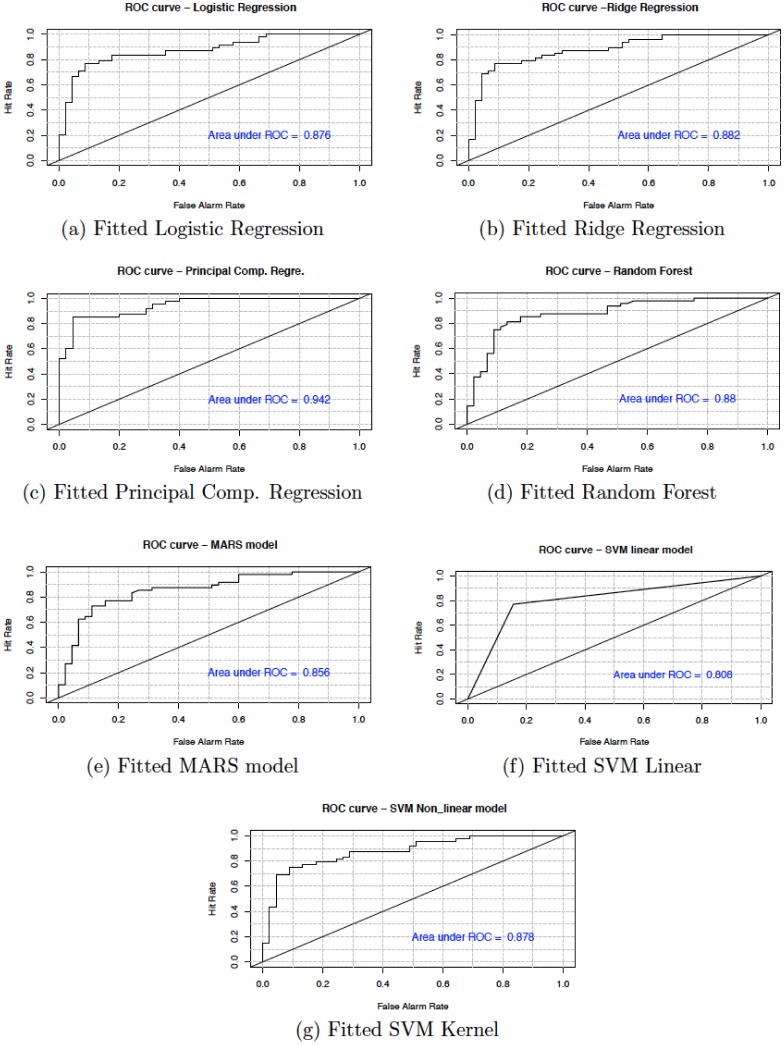
Model evaluation using ROC curve for heart disease data.

In this section, we present the accuracy of our predictive models used in the study. Table 6 shows the sensitivity, specificity, accuracy and confidence interval with 95% significance level for both cancer and heart disease data. Here, sensitivity is the True Positive Rate or the proportion of identified positives among the cancer or heart disease-positive population. Specificity measures the True Negative Rate (TNR) that is the proportion of identified negatives among the cancer or heart disease-negative population. We plotted the ROC curve between True Positive Rate (*Y*-axis) and False Positive Rate (*X*-axis) (see Figures 7 and 8). In these figures, the diagonal line represents the threshold (0.5) of ROC curve. We see that the area under the curve approaches to 1 for the logistic regression (0.998) and random forest (0.997) in cancer data and, for principal component regression (0.942) in heart disease data.

**Table 6.** Prediction accuracy for cancer data.

Models	Sensitivity (%)	Specificity (%)	Accuracy (%)	Conf. Interval (%)
LGR-L_1	92.19	98.94	96.20	(91.92–98.59)
LGR-L_2	83.56	99.36	94.32	(90.49–96.49)
PCR	80.82	1.000	93.89	(89.96–96.62)
RF	97.26	97.44	97.38	(94.38–99.03)
MARS	94.52	98.08	96.94	(93.80–98.76)
SVM-linear	94.54	98.06	96.94	(93.80–98.76)
SVM-nonlinear	93.42	98.69	96.94	(93.81–98.76)

**Table 7.** Prediction accuracy for heart disease data.

Models	Sensitivity (%)	Specificity (%)	Accuracy (%)	Conf. Interval (%)
[0.5ex] LGR-L_1	77.36	90.00	82.20	(73.57–89.83)
LGR-L_2	91.11	72.92	81.72	(72.35–88.92)
PCR	95.56	81.25	88.17	(79.82–93.95)
RF	78.43	88.10	82.80	(73.57–89.83)
MARS	84.44	75.00	79.57	(69.95–87.23)
SVM-linear	77.55	84.09	80.65	(71.15–88.11)
SVM-nonlinear	78.00	86.05	81.72	(72.35–88.98)

## 5. Conclusion

In this study, we analyzed five different machine learning techniques namely; Logistic regression with *L*_1_ and *L*_2_ regularization, Principal component regression, Random forest, MARS model and SVM model. The objective is to use these algorithms to predict the presence of breast cancer and heart disease in patients.

We improved the model for each technique by removing the non-significant variables through cross-validation and using different tuning parameters. Once the predictive models were built, we checked their efficiency in the diagnosis and prognosis of disease using test data. In order to obtain the most efficient model, we compared the prediction mean squared error and miss-classification rate among these models (see Table 5). We also compared the prediction accuracy, sensitivity and specificity to find the best classification accuracy (see Tables 6 and 7).

Additionally, from the plot of the variable importance, we also conclude that a patient suffering from breast cancer are highly affected by the uniformity of cell size (*Cell.size*), bare nuclei (*Bare.nuclei*), uniformity of cell shape (*Cell.shape*), and bland chromatin (*Bl.cromatin*). We also observed that heart disease is highly affected by the chest pain (*cp* ), maximum heart rate (*thalach*), number of major vessels (*ca*), Thalassamia (*Thal*) and slope of the peak exercise ST segment (*slope*) of the patient (see Tables 3,4 and Figures 3,4).

We have shown that the selection of the top variables, based on the variable importance of models, will help researchers to quickly identify specific variables which are relevant to causing breast cancer and heart diseases. The results show that all the techniques described are very efficient to describe and predict the breast cancer and heart disease data. However, based on the area under the curve of the receiver operating characteristic (ROC) we observe that the RF and PCR provides the best fit to the breast cancer and heart disease data. We recall that the ROC curve is a graphical plot that illustrates the diagnostic ability of a binary classifier system as its discrimination threshold is varied.
